# An evaluation of the Kem-O-Mat programmable discrete analyser

**DOI:** 10.1155/S1463924679000420

**Published:** 1979

**Authors:** Geoffrey C. Seymour

**Affiliations:** Division of Clinical Chemistry Northwick Park Hospital, and Clinical Research Centre Harrow Middlesex HA1 3UJ UK


the n-hexane was sampled. This reduced the carry over almost completely for the more volatile benzene constituent (to 0.2% of the original concentration). There was not sufficient removal of the naphthalene and anthracene however, (to 1.6% and 4.7% respectively).

The addition of a rinse vial containing pure mobile phase was then tried. The complete cycle was, therefore: sample; purge; rinse; purge; blank. With only one rinse between samples, all measurable traces of both benzene and naphthalene were removed but a small amount (< 0.1%) of anthracene was still detected. The analyses of the blank samples were made at maximum sensitivity, eight times more sensitive than for the sample. By a small change in the program, the number of rinses from the same vial was increased to three. This successfully removed all traces of carry-over into the blank sample.

Figure 6 illustrates the results obtained using this combination of purging and multiple rinsing. A blank injection, to demonstrate the cleanliness of the system, was followed by an injection of a mixture of mesitylene, pentamethyl, benzene and fluoranthene. After purging, rinsing and purging again, a second sample, this time the benzene, naphthalene, anthracene mixture, was injected. No trace of contamination by the constituents of the first mixture (which have different elution times to the constituents of the second mixture) can be seen in the second chromatogram.

### Conclusions

The automatic liquid chromatograph described in this paper is a reliable and flexible instrument for both routine and research applications. Its simple, straightforward design and construction, incorporating a high proportion of readily available components, has resulted in low hardware costs, while the flexibility of software control allows a good performance to be achieved.

We have shown that it is capable of repetitive sampling with a precision of 1% relative standard deviation, and effective rinse and purge routines can be readily constructed to ensure that all traces of prior contaminants are removed from the system. The accuracy of the data handling algorithm ensures the integrity of the quantitative results even for those samples which are poorly resolved.

We have demonstrated its use with procedures which would probably satisfy most users; one of the outstanding advantages of software control is, of course, the ease with which procedures can be adapted or supplemented in order to cope with new tasks. This is exemplified by the column switching application [2] which uses a peak detection algorithm developed from the data handling program used in this general purpose liquid chromatograph.

### REFERENCES

[1] Willmott, F.W. and Mackenzie, I. *Analytica Chimica Acta,* (1978) **103,** 401.

[2] Willmott, F.W., Mackenzie, I. and Dolphin, R.J. in Schomburg, G. and Rohrschneider, L. (Editors), "Chromatography 1978", Elsevier, p 151.

[3] Brouwer, G. and Jansen, J.A.J. *Analytical Chemistry,* (1973), **45,** 2239.

[4] Anderson, A.H., Gibb, T.C. and Littlewood, A.B., *Analytical Chemistry,* (1970), **42,** 434.

[5] Savitzky, A. and Golay, M.J.E., *Analytical Chemistry, (1964),* **36,** 1627.

[6] Steiner, J., Termonia, Y. and Deltour, J. *Analytical Chemistry,* (1972) **44,**1906.

### An evaluation of the Kem-O-Mat programmable discrete analyser

**Geoffrey C. Seymour**

*Division of Clinical Chemistry, Northwick Park Hospital, and Clinical Research Centre, Harrow, Middlesex, HA1 3UJ, UK.*

This evaluation extended over a period of three months and follows the recommendations of Broughton *et al.* [1.2].

### The instrument

The Kem-O-Mat (Coulter Electronics Limited*) is a calculator-controlled single channel discrete analyser which may be used for either kinetic or endpoint measurement analyses. The throughput of the instrument varies with the methodology, but can be a maximum of 110 samples/hour, at 37°C, in the kinetic mode and 180 samples/ hour in the endpoint mode.

Results are calculated automatically and are printed in the units of choice. The data are checked before calculation to ensure that linearity limits have not been exceeded and that the initial reagent absorbance was within predetermined limits. The data for non-linear analyses, in the kinetic mode, are printed in absorbance units. The operator has the option to print any or all absorbances since all data are stored in the calculator's memory and can be recalled at the end of the analysis run.

All the key instrument functions are continuously monitored by the calculator and fault warnings are given by an audible alarm and printed message.

There is a comprehensive range of methodologies available for the instrument for which the manufacturer will supply the preprogrammed cassettes and the reagent packs, or suggest a supplier for the latter. The user has the facility to develop his own analytical systems on the instrument and prepare his own programmed cassettes.

### Description

The Kem-O-Mat consists of three modules in the air bath version (Figure 1) and four modules in the water bath version. The air bath version was evaluated in this study. Its three modules are a control module to which is fitted the analyser module while the calculator is connected to the control module by electric cable. The control and analyser modules together occupy 590 x 600 mm of bench space. The calculator requires 550 x 365 mm of space. A single 99-132V or 198-264V 50 Hz mains socket supplying 550 VA is the only service required.

The control module contains most of the operational logic, the pump mechanisms and a LED display of the

*Coulter Electronics Ltd., Coldharbour Lane, Harpenden, Herts, UK.*

photometer output in absorbance units. The logic is hard-wired and arranged mainly on plug-in circuit boards.

The analyser module contains the dispensing arm mechanisms and the carousel unit. The carousel, which fits over the filter/photodetector unit, carries up to 32 sample cups round its rim .and up to 32 reaction cuvettes adjacent and concentric to the sample cups. The reaction cuvettes sit within the air bath which is capable of operating at ambient, 30°C and 37°C.

The calculator is a Tektronix 31 programmable model which incorporates a thermal printer and cassette read-write facility, thus making it a versatile general purpose calculator when not engaged in its Kem-O-Mat function.

**Operation**

Having switched the instrument on, and selected the correct bath temperature, the appropriate chemistry cassette is placed in the calculator. The starting block of the operating program is fed into the memory from the tape; this takes about 45 seconds. The printer will then identify the test and start a dialogue with the operator requiring keyboard entries giving batch number, number of cuvettes or selected samples and concentration of the standard, where necessary. Having supplied these data, the calculator will print a check-list to remind the operator to perform certain non-programmable functions before the analysis can proceed. The routine includes checking that the correct bath temperature, dispenser setting, and filter have been selected, and also that the analyser and control modules have been correctly set up. Most of these details would normally be checked, by reference to the method sheet, prior to starting the run, but it serves as a useful reminder to the operator. After the checks a single keyboard entry starts the analysis, everything being totally automated from this stage to the point of printing the results.

The first stage of the analysis is the dilution cycle in which the programmed volume of sample is aspirated into the sample probe and then discharged into the reaction cuvette with the programmed volume of diluent. This process is repeated for the selected number of samples. The diluent can be preheated in the sample arm, as indicated by a light on the control unit. The next cycle then depends on the specified program but the order may vary depending upon the chemistry requirements.

Analysis in the endpoint mode involves taking two absorbance readings on every sample, the first reading acts as a sample blank and the second is the final absorbance of the reaction. The first position in the carousel is always distilled water which serves as a reagent blank. The second position is normally a standard but if a molar absorptivity is used for calibration it can be a sample. The initial absorbance value of each cuvette is multiplied by the dilution factor and this corrected absorbance is then subtracted from the final absorbance for each cuvette; from this value is subtracted the corrected absorbance of the reagent blank. The corrected absorbance is finally multiplied by a calibration factor derived by applying the same calculation to the standard and multiplying by the standard concentration.

In the kinetic mode an alternative scheme of data processing is used. Distilled water is again placed in the first position of the carousel, to serve as a reagent blank, with samples in subsequent position. After the dilution, reagent addition and incubation cycles the absorbance of each cuvette is monitored at each cycle for 5 consecutive cycles (64 seconds/cycle).

Calculations are based on the assumption that the analysis follows a first order linear reaction. Calculation of a result will only occur if 3 or more consecutive absorbances can be used to determine the linear regression, otherwise the result is flagged as being non-linear. Final calculation then corrects for the time constant and the molar absorptivity of the coenzyme/chromogen. Any blank value is subtracted and the result printed, showing which absorbance points were used in the calculation and the absorbance values for reactions considered to be non-linear.

In both analytical modes the operator has the facility to print all the absorbance values taken on all designated

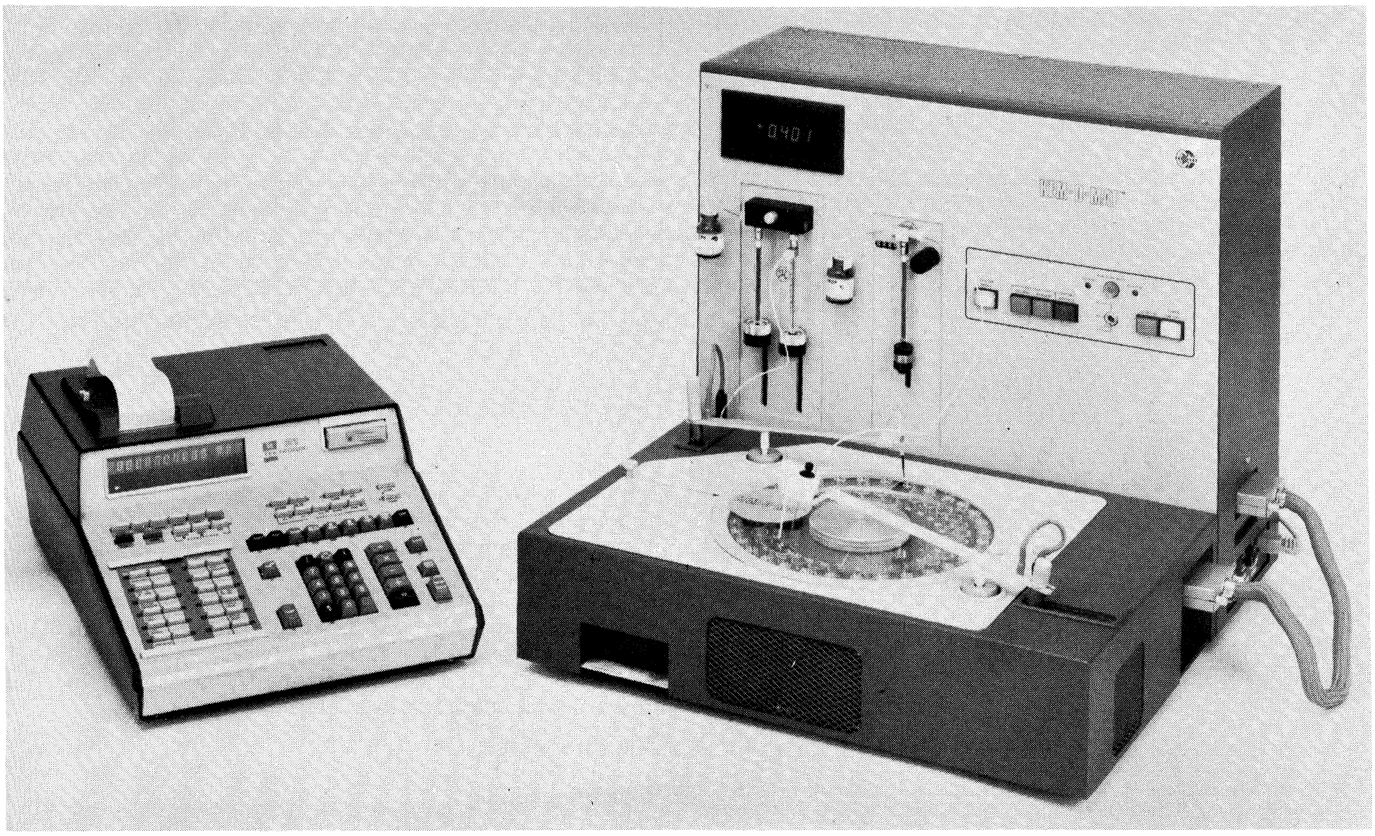

*Figure 1 The Kem-O-Mat (air bath version), showing how the analyser and control modules are connected to form a compact unit.*

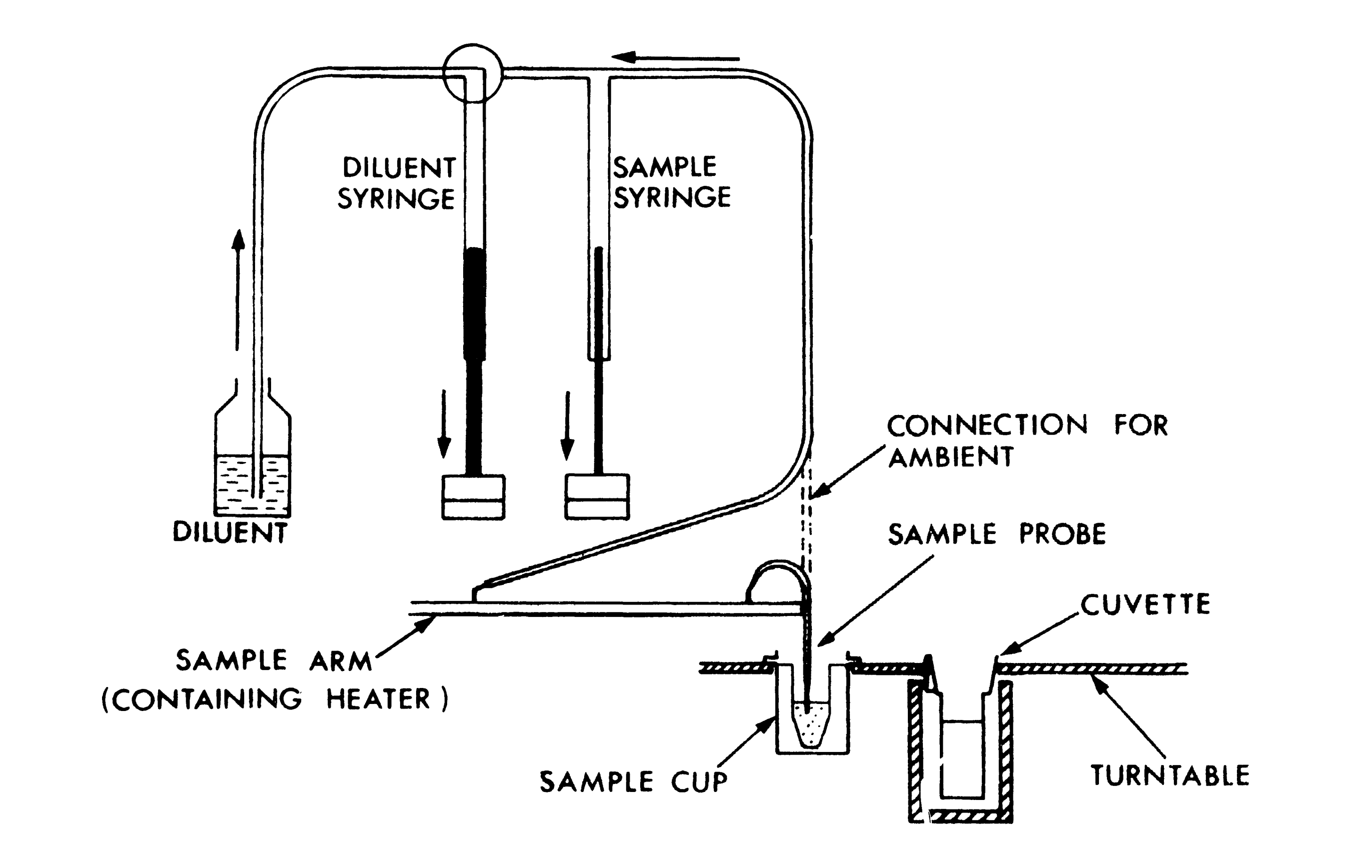

*Figure 2a Diagramatic representation of the sampling process showing how diluent and sample are aspirated simultaneously.*

cuvettes, convert absorbances to concentration units and produce the mean, standard deviation and coefficient of variation of the speciment results.

### The dispenser system

### Mode of operation

The charging and discharging sequences of the Kem-O-Mat are shown diagramatically in Figure 2. The diluent and sample syringes (gas-tight type manufactured by Hamilton Co. Inc.*) are driven in parallel under program control. A manual dispenser switch on the control module is provided for priming purposes allowing for rapid changeover from one chemistry to another while an analysis is in progress.

The second reagent addition is performed by a similar motor-driven Hamilton syringe but in this instance the volume to be dispensed is dialled up on a calibration scale. The same valve arrangement is used as on the diluent syringe with a manual dispenser switch on the control module which allows for priming/recycling of the reagent.

### Precision

The precision and accuracy of the diluent syringe was determined at 3 levels (800μl, 500μl and 250μl) by weight of water dispensed. The sample cups were left empty and the reactio cuvettes weighed before and after dispensing the required volume (see Table 1a).

The precision of the sample syringe was determined at 3 levels (20μl, 10μl and 2.5μl) by the aspiration of an aqueous solution of $^{125}I$ albumin. The sample was dispensed, with 400μl of water, into a disposable sample cup such that the diluted sample had an activity of approximately 10,000 counts per 100 seconds. The sample cup was then capped and read in a Wallac gamma counter (LKB Instruments Ltd+)

** Hamilton Co. Inc., Bonaduz, Switzerland.*

*+L.K.B. Instruments Ltd., S.Croydon, Surrey,England.*

**Table 1 Precision of the dispenser**

| | Nominal volume μl | n | CV% | Mean volume of water dispensed μl |
|---|---|---|---|---|
| Diluent syringe | 800 | 20 | 0.17 | 798 |
| | 500 | 20 | 0.23 | 500 |
| | 250 | 20 | 0.24 | 257 |

| | Nominal volume μl | n | CV% uncorrected for counting error |
|---|---|---|---|
| Sample syringe | 20.0 | 20 | 1.2 |
| | 10.0 | 20 | 1.4 |
| | 2.5 | 20 | 1.2 |

until 20,000 counts had been accumulated. The coefficient of variation (CV) values shown in Table 1b are uncorrected for counting error, which is approximately 0.7%, thereby giving a CV better than 1%.

### Carry-over

The carry-over was investigated according to the recommendations of Broughton et al [2] using alternate groups of specimens with 3 high and 3 low concentrations of analytes. It was determined for each of the assays used in the evaluation for which volumes of serum aspirated were between 5μl and 30μl and diluent volumes between 370μl and 500μl. Carry-over varied between -0.2% and +1.0% (mean 0.6%). As the percentage interaction was less than 2%, cross contamination was not determined.

### The photometric system

### Mode of operation

Light from the tungsten filament lamp passes through an

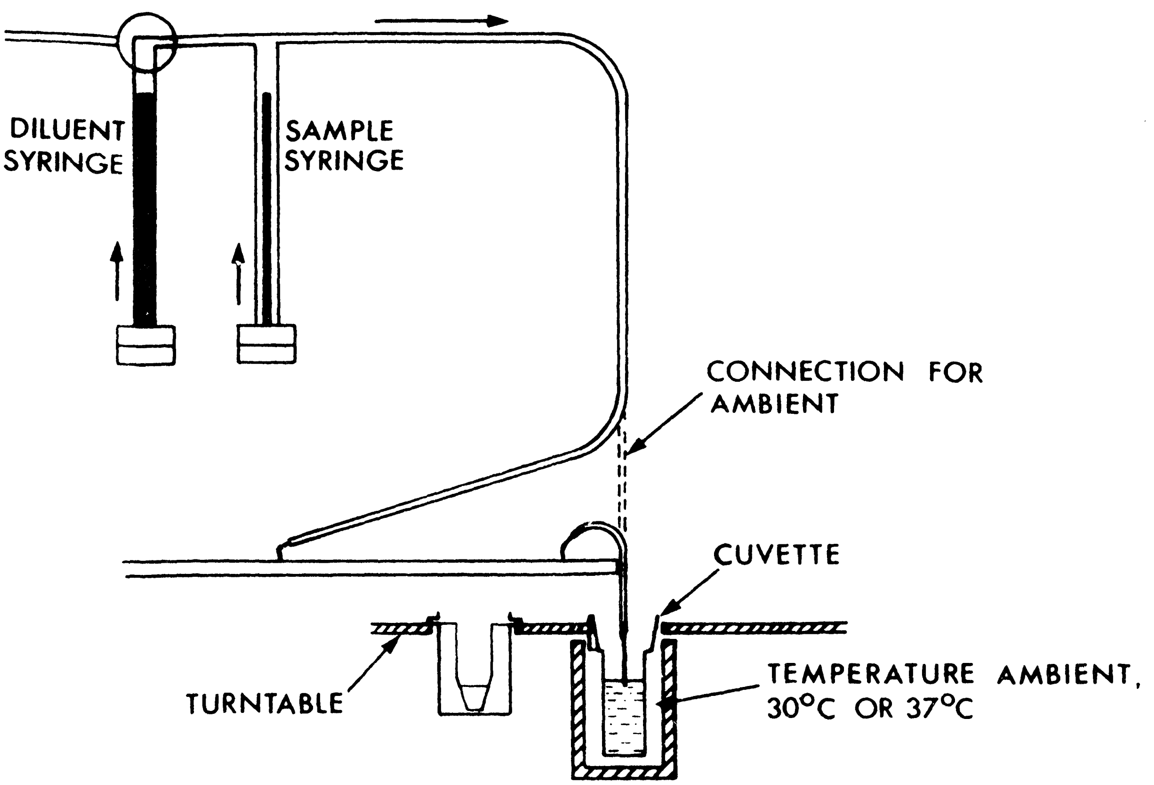

*Figure 2b Diagramatic representation of the dispensing process. The dispensing stroke of the diluent syringe is programmed to finish after that of the sample syringe.*

aperture in the side of the incubation bath and thence through the cuvette to the filter and photomultiplier which are static at the centre of the turntable. Light is also allowed to fall onto a reference photomultiplier tube which is adjacent to the light source and has a fixed reference filter.

The calculator sends a READ command to the control module when the output from the measurement photomultiplier is compared with that from the reference multiplier to produce an absorbance measurement which is displayed on the control module readout. The calculator then generates a *transfer to memory* command and the value of absorbance displayed is stored in the appropriate calculator memory, as determined by the position number of the sample cup in the turntable. The sample cup position number is detected by a code reader which senses the turntable position. At the end of the analysis a *calculate* command is issued. The result is then computed for each sample and the result is printed out against the sample cup number.

**Interference filters**

Filters can be obtained for eleven specified wavelengths over the range 340-700nm; the instrument is normally supplied with filters of 340nm, 405nm and 500nm. Additional 575nm and 600nm filters were supplied with this instrument.

The absorbance spectrum of each filter was recorded using an SP8000 recording spectrophotometer (Pye Unicam Ltd$^{+}$). The observed transmission maxima were all within 0.5nm of the nominal wavelength. The manufacturer claims a half bandwidth of 8nm at 340nm and 9-11nm at all other wavelengths. These figures were checked and found to be within the manufacturer's specification for the 5 filters supplied.

**Linearity and precision**

The linearity of the photometer was determined using acidified aqueous solutions of potassium dichromate. A series of 10 dilutions was prepared with absorbance levels ranging from 0.01 to 2.50 at 340nm; the calibration plot showed a straight line passing through the origin. The same solutions were used to determine the within-batch and between-batch precisions.

Duplicate 500µl amounts of each solution were placed in cuvettes and, after a warm-up period of 30 minutes, absorbance readings were taken 20 times over 30 minutes by manually advancing the carousel. Between-cuvette precision was determined on cuvettes from the same injection moulding and between four different injection mouldings. Of the four moulds, only two produced cuvettes of sufficiently high quality to be included in these studies. All poor cuvettes were returned to Coulter Electronics and replaced with those from the two good mouldings.

Cuvettes were filled with distilled water and absorbance readings taken as in the previous experiment. This water was then carefully removed, the cuvettes rinsed twice in the potassium dichromate solution to be read and readings taken on the third filling as in the previous experiment. The reason for this mode of operation is that the Kem-O-Mat never measures the absorbance of an empty cuvette, where optical characteristics are different from those of a filled cuvette. Cuvettes are emptied and filled *in situ* because they are held rigidly in the carousel by a frosted top, with a location key on top of the cuvette. If the cuvette is removed and then replaced in the carousel it will not always give the same absorbance.

Results from these three experiments are shown in Figure 3 individual points are not shown to prevent confusion.

The performance of the photometer overall would appear to be adequately precise over the absorbance range of 0.1 to 2.5 units at 340nm.

**Stability**

The absorbance of a cuvette filled with potassium dichromate solution and then sealed with parafilm was measured

$^{+}$*Pye Unicam Ltd., York Street, Cambridge, CB1 2PX, England.*

**Table 2 Methods used on the Kem-O-Mat**

| Method | Reagents and reference | Sample volume µl | Total volume µl | Mode | Wavelength nm |
|---|---|---|---|---|---|
| Alkaline phosphatase | † Alkaline Phosphatase reagent twin set pack: Bowers et al [4] | 15 | 430 | Kinetic | 405 |
| Cholesterol | † Enzymatic cholesterol reagents single vial pack: Allain et al [5] | 10 | 500 | Endpoint | 500 |
| Creatine kinase | * CK 'activated' U.V. method: Bergmeyer [6] | 10 | 440 | Kinetic | 340 |
| Glucose | † Glucose (Hexokinase) reagent twin set pack: Peterson et al [7] | 5 | 500 | Endpoint | 340 |
| Triglycerides | † Triglycerides reagents twin set pack: Bucolo et al [8] | 10 | 540 | Endpoint | 340 |
| Uric acid | * Urica-quant uric acid test combination: Kageyama [9] | 30 | 470 | Endpoint | 405 |
| Uric acid | + Spinchem Uric Acid reagent: Haeckel [10] | 30 | 500 | Endpoint | 340 |

*† Coulter reagents, manufactured by Calbiochem Ltd.*
** Boehringer Mannheim Corporation*
*+ Smith Kline Instruments, Inc. (SKI)*

*in situ* 50 times over a period of eight hours, at 37°C. The mean absorbance was 0.043 ± 0.003 (CV 2.5%). No systematic drift was discernible.

**Temperature control**
The instrument is supplied with either an air bath which is integral with the analyser unit, or with a water bath which is connected as a separate module; the instrument under evaluation had an air bath which consists of an annular metal well in which the cuvettes revolve, the heating and sensing elements being contained within this metal well. The temperature regulating circuits are housed within the control module with separate adjustments for 30°C and 37°C which are factory set, checked on installation and recommended not to be adjusted by the user.

**Test conditions**
Measurements were taken using a thermistor probe assembly (Yellow Springs Instrument Co.+ No. 44005 composite probe) connected to a chart recoder.

The design of the Kem-O-Mat made it impossible to take temperature recordings with the carousel cover plate in position, thus the stability measurements were made by pushing a cap onto a cuvette containing 500µl of water (a special plate is now available from the manufacturer for this purpose). The probe was then inserted through a small hole in the cap allowing the probe to rotate with the carousel. Measurements of temperature variations during operation were done with the probe held against the inside of the cuvette with a spring placed at the top of the cuvette, positioned so as not to interfere with the dispensing and mixing mechanisms. The carousel was fully loaded with cuvettes and sample cups throughout the experiment. The chart recorder was set at 5°C full scale deflection.

**Stability**
By use of the temperature control program on the 'instrument-check' cassette it was possible to run the instrument in the incubation mode continually. The temperature variation over 8 hours was 36.8 ± 0.13°C with no observable influence caused by draughts induced by an open door approximately one metre from the instrument. The ambient temperature rose from 19°C to 24°C over the period, with no apparent drift in the temperature of the cuvette contents.

*+Yellow Springs Instrument Co., Yellow Springs, Ohio 45387, USA*

**Variations during operation**
The time taken for the contents of a cuvette to reach 37°C from start up was 32 minutes for an ambient temperature of 20°C; the manufacturer suggests that at least 30 minutes be allowed for warm up when starting from ambient. The reaction bath temperature indication light started flashing much earlier than this, denoting that although the metal heating block may have reached the desired temperature the cuvette contents had not. This feature should be taken into consideration when any change in working temperature is required.

The thermistor assembly was used to monitor the temperature variation during instrument operation. Output was continually monitored with the probe placed in a cuvette at position 3 in the carousel.

A test program was developed and used to operate the instrument under the following conditions: with the preheaters connected to both the sample and reagent arm a specified volume of sample was diluted with a given volume of water; 5 minutes later a known volume of water was added via the reagent dispenser arm. For volumes of 250µl and 500µl of diluent only 20 seconds was required to reach a stabilized temperature; subsequent addition of 100µl of preheated water caused an apparent drop of 0.14°C with return to the original temperature within 20 seconds. With the sample arm preheater disconnected, 6.5 minutes were required for 250µl and 8 minutes for 500µl of diluent to reach 37°C (ambient temperature 20°C).

The manufacturer stresses the importance of having all positions on the carousel filled with cuvettes. When the two cuvettes adjacent to the one containing the thermistor were removed, the temperature in the test cuvette dropped by 1.5°C and maintained the lower temperature.

### Chemistry evaluation

The evaluation followed the scheme of Broughton *et al* [1,2] for precision and linearity. Comparisons of accuracy and precision were made against routine methods employing a variety of analytical techniques and instruments.

**Table 3 Between bath precision : Kem-O-Mat and routine methodology**

| | Kem-O-Mat | | | Routine methodology and reference | Routine method | | |
|---|---|---|---|---|---|---|---|
| | n | Mean | CV% | | n | Mean | CV% |
| Alkaline phosphatase | 20 | 98.1 | 3.00 | * Vickers M-300 discrete analyser | 40 | 105 | 4.10 |
| IU/l | 20 | 171.1 | 1.40 | King and Kind [11] | 40 | 206 | 3.40 |
| | 20 | 312.0 | 1.33 | | | | |
| Cholesterol | 20 | 3.91 | 1.95 | + AutoAnalyser Mk I | 40 | 3.80 | 3.10 |
| mmol/l | 20 | 4.36 | 1.43 | Seymour & Gray [12] | 40 | 5.20 | 3.40 |
| | 20 | 5.28 | 1.18 | | | | |
| Creatine kinase | 20 | 29 | 31.00 | † LKB reaction rate analyser | 40 | 60 | 8.20 |
| IU/l | 20 | 227 | 13.00 | Bergmeyer [6] | 40 | 700 | 9.20 |
| | 20 | 638 | 8.90 | | | | |
| Glucose | 20 | 3.33 | 1.41 | * Vickers D-300 discrete analyser | 40 | 3.8 | 3.00 |
| mmol/l | 20 | 6.10 | 1.51 | Trinder [3] | 40 | 11.4 | 1.70 |
| | 20 | 11.20 | 1.40 | | | | |
| Triglycerides | 20 | 0.42 | 8.36 | + AutoAnalyser Mk II | 20 | 0.40 | 22.70 |
| mmol/l | 20 | 2.28 | 3.19 | Wahlefeld [13] | 20 | 6.00 | 1.80 |
| | 20 | 5.80 | 3.29 | | | | |
| Uric acid (SKI) | 20 | 298 | 4.20 | * Vickers M-300 discrete analyser | 40 | 310 | 3.60 |
| μmol/l | 20 | 353 | 2.77 | Morin [14] | 40 | 550 | 2.50 |
| | 20 | 478 | 1.07 | | | | |

* *Vickers Ltd., Priestley Road, Basingstoke, Hants.*
\+ *Technicon Instruments Ltd., Houndsmills, Basingstoke, Hants, RG21 1BZ*
† *LKB Instruments Ltd., 232 Addington Road, S. Croydon, Surrey. CR2 8YD*

The Coulter reagents are supplied in various packagings, the majority containing lyophilised material stated to be stable for 1 year at 0-4°C. Reconstituted reagents vary in stability; the shortest period is stated to be 48 hours at 4-8°C.

### Analytical methods and comparison studies

The methods and conditions used on the Kemo-O-Mat are outlined in Table 2. Aqueous standards were used for the Urica-quant uric acid test and the Coulter hexokinase methodologies. The cholesterol method required an assayed control serum whilst the other chemistries used molar absorptivities for calculation of results.

Reagents for alkaline phosphatase assays were supplied by Coulter as a two-vial reagent pack. The sample is diluted with 2-amino-2-methyl-1-propanol and incubated at 37°C. The substrate, 4-nitrophenylphosphate, is then added as the starter reagent and the liberated 4-nitrophenol measured kinetically at 405nm. Activity is determined using the molar absorptivity of the product.

Cholesterol reagents were supplied by Coulter as a single-vial pack containing cholesterol esterase, cholesterol oxidase, peroxidase and the Trinder [3] chromogen. The sample is diluted with distilled water and reagent is added via the starter syringe. Absorbance measurements, at 500 nm are taken 12 seconds and approximately 10 minutes after addition of the starter reagent. Results are calculated from a standard incorporated with each batch of analyses.

The creatine kinase method cassette was prepared locally using the facility program provided by Coulter Electronics. Boehringer* reagents were used for this assay in order to simplify comparison with the method currently in use in the laboratory. The sample is diluted with buffered substrate and incubated at 37°C for 5 minutes; creatine phosphate is added as the starter reagent and the initial absorbance is taken 2 minutes later. Increase in absorbance at 340nm is monitored for the next five revolutions, and the activity calculated.

Glucose (hexokinase) reagents were supplied by Coulter as a two-vial reagent pack, although a single-vial reagent is available. Sample is diluted with buffered substrate and the initial absorbance at 340 nm monitored; NADP is then added as starter reagent and a second absorbance measurement taken approximately five minutes later. Results are calculated from a standard included with each batch of analyses.

Triglyceride reagents were supplied by Coulter as a two-vial reagent pack. The procedure involves enzymic hydrolysis of triglycerides, phosphorylation of glycerol with glycerol kinase and measurement of the decrease in concentration of NADH in a coupled reaction. The diluent contains all the reagents except for the glycerol kinase which is used as the starter reagent. The sample is incubated with the diluent for approximately ten minutes at 37°C and the absorbance at 340nm is monitored with subsequent addition of glycerol kinase; a final absorbance reading is taken approximately six minutes later. Triglyceride concentration is calculated using the molar absorptivity of NADH.

The uric acid method originally recommended by Coulter was Urica-quant, an assay kit supplied by Boehringer Mannheim using the method of Kageyama [9]. Correlation of results from this method compared with the routine method gave a slope of 0.76 with an intercept of +126μmol/l and a correlation coefficient of 0.96; this was shown to be due to a non-linear calibration curve obtained with the Urica-quant technique. Straight lines could not be drawn through calibration curves produced on the Kem-O-Mat using either serum or aqueous standards. Kem-O-Mat endpoint chemistries are based on a 2 point calibration and therefore any reaction must obey Beer-Lambert's law; for this reason the method was abandoned and fresh trials started using an assay kit supplied by Smith Kline Instruments +(SKI).

The reaction involves conversion of uric acid into allantoin and hydrogen peroxide in the presence of uricase. The hydrogen peroxide is then converted into acetate in the presence of aldehyde dehydrogenase with concomitant formation of NADPH. Reagents are supplied such that only uricase is prepared separately as the starter reagent, the other reagents being incorporated in the diluent. Sample is dispensed with diluent, incubated for approximately 3 minutes and the absorbance, at 340nm, taken. Uricase is then

**Boehringer Corporation (London) Ltd., Bell Lane, Lewes, East Sussex, BN7 1LG, England.*

*+Smith Kline Instruments Co. Ltd., Welwyn Garden City, Herts, England.*

**Table 4 Within-bath precision of the Kem-O-Mat**

| | n | Mean | CV% |
|---|---|---|---|
| Alkaline phosphatase IU/l | 20 | 172 | 0.63 |
| Cholesterol mmol/l | 20 | 3.83 | 1.7 |
| Creatine kinase IU/l | 20 | 39.4 | 7.1 |
| Glucose mmol/l | 20 | 4.00 | 0.56 |
| Triglycerides mmol/l | 20 | 2.33 | 1.4 |
| Uric acid (SKI) μmol/l | 20 | 299 | 1.5 |

added and incubation continued for a further 6 minutes, after which the final absorbance is read. Uric acid concentration is calculated using the molar absorptivity of NADPH.

The methods and analytical techniques used for the comparison studies are indicated in Table 3.

### Precision

Within-batch precision was assessed for each determination by performing 20 replicate measurements on a control serum in a single run (Table 4). Between-batch precision was assessed over 20 working days, performing one run per day except in the case of uric acid using SKI reagents, when 20 batches were assayed during 10 working days. One person operated the instrument over this period.

In total, 141 runs were performed, of which 1 was rejected due to an instrument malfunction. Any results, from a kinetic analysis, which were reported as non-linear were not included in any statistical calculations.

The between-batch precision study followed the recommendations of Broughton *et al* [2]. Wellcome+ Unassayed One serum was used for low levels, and Autoset H for high levels of constituents. After reconstitution in accordance with the manufacturer's instructions, a third serum pool was prepared by mixing 2 volumes of the Unassayed One serum with 1 volume of Autoset H serum, giving a specimen with intermediate levels. Portions of all 3 sera were stored frozen (-20°C) and a group thawed each day for analysis. Results were obtained from 20 acceptable runs for each determination. There was no discernible trend in any set of results.

The between-batch results together with data from the comparative methodologies are given in Table 3.

### Relative accuracy

No attempt was made to compare the accuracy of the Kem-O-Mat results with those of reference techniques; results were compared with values obtained using the corresponding current laboratory methodology for which figures for precision and relative accuracy were known.

At least 180 sera from different patients were analysed for each constituent during the period of precision assessment. These specimens were selected at random from the laboratory workload with the minimum amount of delay between the comparative runs. If storage was necessary it was overnight at 4°C in the dark. A regression analysis was performed on each set of results (Table 5), showing acceptable correlations with the routine methods.

### Linearity

For each constituent except creatine kinase, several patient sera with high levels were pooled and subsequently diluted to prepare a calibration curve. A series of aqueous standards was also run for cholesterol, glucose and uric acid, and the linearity assessed by plotting the calibration curve and noting where the slope deviated from linearity.

The results for these determinations are given in Table 6. The aqueous standards for glucose and uric acid (SKI) gave results consistent with serum studies, whereas the aqueous micellar cholesterol standards prepared according to Richmond [15], showed a different reaction time-course to serum; and therefore an assayed control serum was used to calibrate the instrument for this assay.

The observed limits of linearity for glucose and cholesterol were considerably lower than the manufactuer's claims; both limits could be increased by increasing the incubation period.

### Safety

New recommendations covering the electrical safety of hospital laboratory equipment in the UK have recently been published [16]. The instrument was found to comply with these recommendations.

In the analyser module all electronic components, as far as possible, are mounted away from areas of likely spillage and most junction terminal strips are protected by a plastic cover.

service, the casing is easily removed and components are moderately accessible. Most of the electronic parts in the control module are mounted on plug-in printed circuit boards which may be easily removed for service or replacement.

Certain electrical tests recommended in the Electrical Safety Code [16] were carried out and the results are listed in Table 7. The instrument performed satisfactorily at ± 10% of 240 Va.c. (i.e. 216 Va.c. and 264 Va.c.).

Two features could give rise to problems. The diluent container is mounted directly over the heater plug for the sample arm and spillage is possible. Components and optics may become dusty due to a fan which is in operation when

+*Wellcome Reagents Limited, Beckenham, Kent, BR3 3BS, England.*

**Table 5 Correlation of results : Kem-O-Mat (x) v Routine Methodology (y)**

| | Range of values | | n | $\bar{x}$ | $\bar{y}$ | Regression equation | r |
|---|---|---|---|---|---|---|---|
| Alkaline phosphatase IU/l | 21.8 | 444 | 378 | 104.3 | *82.8 | y = 0.67x + 11.9 | 0.979 |
| Cholesterol mmol/l | 2.0 | 12.4 | 363 | 5.92 | 6.15 | y = 1.02x + 0.15 | 0.978 |
| Creatine kinase IU/l | 6 | 1560 | 378 | 170.3 | 176.0 | y = 0.98x + 10.3 | 0.996 |
| Glucose mmol/l | 1.5 | 23.8 | 382 | 5.49 | 5.75 | y = 1.02x + 0.14 | 0.992 |
| Triglycerides mmol/l | 0.37 | 7.35 | 195 | 2.00 | 2.18 | y = 1.05x + 0.07 | 0.996 |
| Uric acid (SKI) μmol/l | 138 | 937 | 184 | 293.0 | 292.6 | y = 1.01x – 5.32 | 0.985 |

* *Results in King-Armstrong units/100ml x 7.1 to convert to International Units.*

**Table 6 Linearity of the methods**

| | Claimed upper limit of linearity | Observed upper limit of linearity |
|---|---|---|
| Alkaline phosphatase IU/l | 400 at 30°C | 400 at 37°C |
| Cholesterol mmol/l | 13.0 | 9.5 |
| Creatine kinase IU/l | – | 1,000 at 37°C |
| Glucose mmol/l | 28.0 | 22.0 |
| Triglycerides mmol/l | 5.60 at 30°C | 5.60 at 37°C |
| Uric acid (Boehringer μmol/l Urica-quant | 1190 | (non linear) |
| Uric acid (SKI) μmol/l | – | 1500 |

**Table 7 Results of electrical safety checks**

| Test | ESCFHLE † Paragraph ref. | Result |
|---|---|---|
| Leakage current | 182 | <10 micro amps in both directions |
| Insulation resistance | 95 | >30 meg ohms at 500 V d.c. |
| Earth continuity | 61 | ≯0.4 ohms * |
| Fusing Factor | 146 | 1.17 |

† *Electrical Safety Code for Hospital Laboratory Equipment [16].*
* *This test was not carried out at 25 amps as recommended for fear of causing damage to the Kem-O-Mat.*

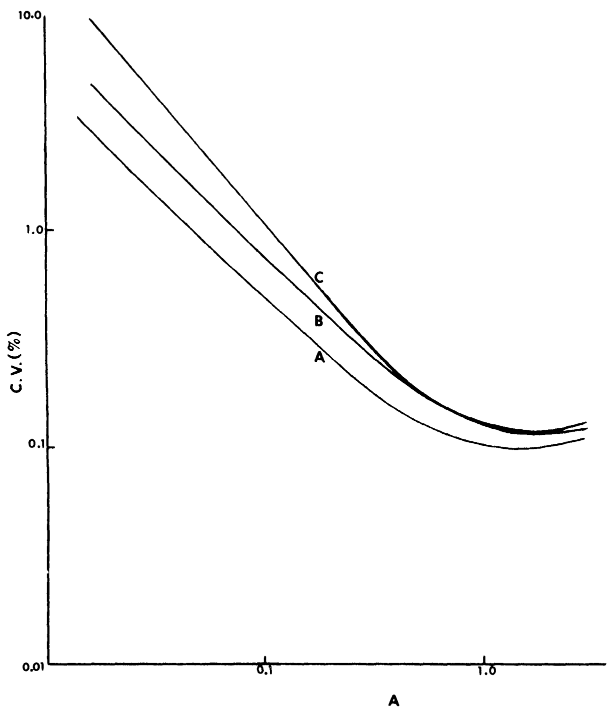

*Figure 3. Precision of the photometer*
*A Within Cuvette*
*B Between cuvettes from the same mould*
*C Between cuvettes from different moulds*

the power is on and there is no filter over the air intake grill.

The design of the instrument is such that there is no danger to the operator of a mechnically induced injury. There is however a potential danger in the design of the sample and reagent dispenser arms. It is possible for the instrument to operate with either or both of these arms in the vertical position with the possibility of reagent being sprayed across the instrument, operator and laboratory.

A check on aerosol formation around the dispensing and stirrer mechanisms was made using a non-pathogenic bacillus species. The instrument was run for 2 complete cycles both dispensing and stirring the microbial suspension with settle plates placed around and in close proximity to both mechanisms. No bacteria could be grown on any of these plates. This was somewhat surprising, with regard to the stirrer mechanism because of its vigorous motion even after leaving the cuvette. It was concluded that serum aerosols are unlikely to be a hazard.

### Dependability

During the 12-week evaluation period the instrument was switched on for 460 hours and was actually operative for approximately half of this time.

A fault on the rotary solenoid of the diluter arm caused the rejection of 1 batch of assays and the loss of 1 day's work awaiting an engineer. The fault was found to be due to an assembly error with a countersunk screw burred and catching on the rotary solenoid mechanism. Another intermittent problem occurred in the loading of data from a cassette into the calculator; occasionally there would be a mis-read which was detected in the dialogue section. The original calculator was replaced but the error still occurred. This only happened on five occasions during the evaluation and was remedied by reading the relevant block tape into the memory again, requiring a maximum of an additional 45 seconds. The manufacturer says that this was due to a tape error which has now been corrected.

### Costing

In January 1979 the purchase price of the Kem-O-Mat in the United Kingdom was £13,350 (excluding Value Added Tax).

The cost of the pasteur pipettes used for dispensing the sera into the sample cups, was not considered. The diluent dispensing system has a dead volume of 1.0ml whilst that of the reagent system is 0.7ml. The design of the reagent dispensing system makes effective priming/recycling of the reagent possible with minimal losses; this is not so with the diluent system and loss of at least the 1.0ml dead volume must be assumed. There are several factors which influence the cost-per-test analysis; these include the packaging of reagent by Calbiochem Ltd.+, the number of vials used in any batch of assays, and whether unused reagent is stored until the next day. For this reason, the manufacturer's costs for reagents, assuming total usage, are given in Table 8 together with the costings found from practical experience with the instrument. The latter assumes that only one set of reagents per test is reconstituted that the dead volume is taken into account and that no unused reagent is stored. The costing of the creatine kinase and uric acid reagents are only given as found in practice, since alternative reagents were used to those supplied by Coulter. The costings in Table 8 refer only to reagents; other consumable costs are as follows:-

Kem-O-Mat cuvettes. . . . . . . . . . . . . . . 1.60p each
Either: 0.5ml sample cup. . . . . . . . . . . . 0.88p each
Or: 2.0ml sample cup. . . . . . . . . . . . . . 0.65p each

+*Calbiochem Ltd., San Diego, California, USA.*

**Table 8 Reagent costing exclusive of depreciation, labour and consumables**

| Method | Rack type | Manufacturer estimated No. of tests per vial | Manufacturer estimated cost per test (p) | Actual tests per vial | Actual cost per test (p) |
|---|---|---|---|---|---|
| Alkaline phosphatase | Twin set pack | 36 | 3.3 | 25 | 4.8 |
| Cholesterol | Single vial | 30 | 10.6 | 23 | 13.8 |
| Creatine kinase | Two reagents | – | – | 64 | 11.8 |
| Glucose | Single vial | 30 | 4.0 | 23 | 5.2 |
| Triglycerides | Twin set pack | 31 | 8.0 | 29 | 8.6 |
| Uric acid | Two reagents | – | – | 44 | 10.0 |

The cuvettes were used only once as recommended by the manufacturer, one cuvette being used for one assay.

Labour costs can be estimated from the average throughput time, during the evaluation which was better than one result per minute for one operator.

### Overall assessment of the system

### Operation

An operating manual is provided with the instrument. This has two main sections, one for the Kem-O-Mat itself and one for the Tektronix 31 calculator. For convenience the cover of the combined manual is designed to accept 9 programmed cassettes. Several sections of the manual have been revised since the first issue in February 1977 but a few minor errors in specification are apparent.

In general the manual is well written and easy to understand; there are sections on fault finding and the method of calculating results. No electrical wiring diagrams are included and there are no statistical data for the chemistries supplied. An 'instrument check' cassette tape with the relevant operating instructions is supplied and serves as a good check on most of the mechanical and logical functions of the instrument.

Ease of operation with minimal instruction is mainly facilitated by a dialogue through the printout from the calculator, and a series of error messages that are either given as an audible alarm plus printout or as a display on the calculator. Changing from one chemistry to another can be accomplished, to a large degree, whilst the analyser is performing the current chemistry. Changing to a new working temperature may require a considerable period of time to stabilise at the newly selected temperature. Operation at 30° C requires an ambient temperature no higher than 26°C which may be difficult to maintain in summer (in certain climates) without air conditioning.

According to the manual, an error message of ABS>2.5 is printed when any cuvette has an absorbance of greater than 2.5. This implies that the error message is given on raw data and not after applying any calibration factor.

### The instrument

### Dispenser system

All the early problems with the instrument, experienced before the evaluation period, stemmed directly or indirectly from the dispenser unit. Several designs of sample probe were tested before the evaluation and the one that gave the most satisfactory results was used during the test period; this was constructed of P.T.F.E. impregnated nickel/steel.

The diluent dispenser arm has a pre-heat facility linked to the temperature-select switch. The manufacturer has made various recommendations for its use and at the time of evaluation the heater unit was constantly in use, irrespective of the sample volume. The heating bath was set at 37°C for all six chemistries performed in the evaluation even though the manufacturer recommended 30°C for 2 of the analyses (alkaline phosphatase and triglyceride). This was done for convenience and seems to have had no effect on the results.

The syringe mechanism is difficult to fault, being robust, accurate and precise and is in no way a limiting factor in the overall precision which is achieved.

### Photometric system

The photometer showed no drift over the period of one working day but fluctuations about a mean absorbance level of 2 to 3 milli absorbance units were detectable; this variation is insignificant for endpoint analyses but was the cause of several seemingly slightly non-linear results in the kinetic mode. Whilst this instability is greater than was observed with the Abbot ABA-100+ [17] its contribution to the overall imprecision of the instrument only becomes important with very low absorbance levels.

Before the evaluation, the cuvettes were the cause of poor precision due to excessive static associated with the acrylic moulding material. Serum could be seen to leave the probe at such an acute angle that it did not enter the reaction mixture. A process has since been introduced, during manufacture, which has overcome this problem but there is still an optical problem associated with poor moulding, as discussed earlier, and it is of prime importance that there is available a reliable source of acceptable cuvettes.

The interference filters that were tested conformed to the manufacturer's specifications.

### Temperature control

The temperature stability of cuvette contents was found to be slightly outside the manufacturer's specification and considerably outside the IFCC recommendations for temperature control of measurement of catalytic concentrations of enzymes which is ± 0.05°C from the nominal set-point temperature [18]. These findings may have been due to the inability to take readings with the carousel cover-plate in position. Use of the preheater facility on the diluter and reagent arm meant that reagents could be added at the selected temperature.

### Calculator

The calculator is simple to operate and a minimal amount of maintenance is required. The thermal printer is quiet and the printing clear. Connections are provided for an interface (available from Coulter Electronics) to a computer or standard peripheral.

### Chemistry performance

Cholesterol and glucose (hexokinase) gave good precision with good correlation data but the linear range was well below specification.

The triglyceride assay gave reasonable precision with linearity better than claimed by the supplier; this was due to running the determination at 37°C and not 30°C as suggested. An accountably high reagent blank was found by both the author and supplier. The blank values did not affect precision, as was to be expected, but could markedly affect accuracy. For this reason it was decided to follow the suggestion of Coulter Electronics and calculate the results ignoring any reagent blank. Of the 20 batches run during the evaluation 5 were calculated ignoring the reagent blank.

+*Abbott Laboratories, Queenborough, Kent, ME11 5EL, England*

Since the end of the evaluation the assay has been in routine use and no problem has been experienced, although the reagents were from the same batch. Further clarification of the difficulty is required.

Original studies on the uric acid methodology were performed using the Boehringer Urica-quant kit, as recommended by Coulter, but the correlation studies showed poor agreement with standard techniques due primarily to a non-linear reaction course. This phenomenon had been reported previously in an evaluation study on the Abbot ABA-100 analyser by Bullock *et al* [17]. It was for this reason that a further study on uric acid measurement was performed using an NADP coupled reaction as detailed by SKI. This is a two-reagent method, uricase being the starter reagent, and is based on the oxidation of acetaldehyde by aldehyde dehydrogenase in the presence of NADP. On the Kem-O-Mat the reaction was found to be linear to 1500 $\mu$mol/l showing a good correlation with the routine method.

The creatine kinase assay would appear to give poor between-batch precision, but of the same order as that obtained in the routine laboratory; the difficulty is a reflection of the chemistry and not the functioning of the instruments involved.

The reagent costs given in Table 8, show the best and worst cases of reagent utilisation. The manufacturer quotes figures for total reagent consumption whereas the figures found in use are determined from opening a single vial of reagent; the true reagent cost probably falls somewhere between the two. Some of these costs would appear to be high, but when compared with the costs on other instruments in the author's laboratory a net saving can be made on selected chemistries in which expensive reagents are used.

The Kem-O-Mat was found to give an acceptable performance both during the evaluation period and in subsequent use.

ACKNOWLEDGEMENTS

Thanks are due to: Coulter Electronics Ltd., for the loan of the instrument, the supply of reagents and consumables, and help during the evaluation and preparation of this report; Mr. R.J. Wood for his work in testing the electrical and mechanical safety of the instrument as well as the construction of the thermistor assembly; Mr. B. Hawgood for the supply of the microbial suspension, settle plates and subsequent interpretation of the relevant results; to Dr. M.G. Rinsler, Dr. S.S. Brown and Dr. F.L. Mitchell for encouragement and critical comment of the report.

REFERENCES

[1] Broughton, P.M.G., Buttolph, M.A., Gowenlock, A.H., Neill, D.W. and Skentelbery, R.G., *Journal of Clinical Pathology,* 1969, 22, 278.
[2] Broughton, P.M.G., Gowenlock, A.H., McCormack, J.J. and Neill, D.W., *Annals of Clinical Biochemistry,* 1974, **11**, 207.
[3] Trinder, P., *Journal of Clinical Pathology,* 1969, 22,246.
[4] Bowers, G.N. and McComb, R.B. *Clinical Chemistry,* 1966, **12**, **70**.
[5] Allain, C.C., Poon, L.S., Chan, C.S.G., Richmond, W. and Fu, P.C., *Clinical Chemistry,* 1974, **20**, 470.
[6] Bergmeyer, H.U. *Journal of Clinical Chemistry and Clinical Biochemistry, 1975,* **13**, 507.
[7] Peterson, J.I. and Young, D.S., *Analytical Biochemistry,* 1968, 23,301.
[8] Bucolo, G. and David, H., *Clinical Chemistry,* 1973, **19**, 476.
[9] Kageyama, N., *Clinica Chimica Acta,* 1971,**31**, 421.
[10] Haeckel, R.J., *Journal of Clinical Chemistry and Clinical Biochemistry,* 1976,**14**,101.
[11] Kind, P.R.N. and King, E.J., *Journal of Clinical Pathology,* 1954,7, 322.
[12] Seymour, G.C. and Gray, C.J., *Medical Laboratory Sciences,* 1978, **35**,55.
[13] Wahlefeld,, A.W., in 'Methods of Enzymatic Analysis' Ed. Bergmeyer, H.U., 1974, Adademic Press p. 1831.
[14] Morin L.G., *Clinical Chemistry,* 1974, **20**,51.
[15] Richmond, W., *Clinical Chemistry,* 1976, **11**,1579.
[16] 'Electrical Safety Code for Hospital Laboratory Equipment.' 1977, H.M.S.O., London.
[17] Bullock, D.G., Badham, L.P. and Wilding, P., 'An evaluation of the Abbott Biochromatic Analyser'. Special Report No. 2. Wolfson Research Laboratories, Queen Elizabeth Medical Centre, Birmingham, England.
[18] Bowers, G.N., Jr., Bergmeyer, H.U., and Moss, D.W., *Clinica Chimica Acta, 1975,* **61**,F11.

### Short Communications

### Automated dissolution rate analysis of iron in some vitamin preparations

**Bo Karlberg and Sidsel Thelander**

Astra Pharmaceuticals, Analytical Control, S-151 85 Sodertalje, Sweden.

Dissolution rate analysis (DRA) is often required in the quality control of slow-release preparations. Manually this type of analysis is very tedious and time-consuming. Shah, et al [1], have summarized the requirements that are necessary on a system for DRA. For instance, the withdrawal of the samples must be made without interrupting the agitation of the solution. In many systems sample acquisition is performed continuously. The test solution is pumped through a filter into a flow cell and then back to the dissolution vessel. This is a convenient arrangement for some applications but it has a severe limitation. If the active constituent does not possess absorption properties within UV or visible regions an additional treatment of the sample is necessary. In this case the sample will be destroyed. The aliquots must therefore be removed at known times and analysed later. Furthermore, they must be so small as to avoid a significant change in the volume of the dissolution fluid.

This paper describes a fully automated system for DRA of iron in some vitamin preparations. The dissolution is determined at one, two and four hours. The system is commercially available* and very versatile since the analytical cartridge can be modified easily. For example, potassium in slow release preparations can be determined by using a flame photometer detector [2].

### Materials and method

### Reagents

All chemicals should be of Reagent grade.

*R 1: Hydroxylamine in 2 M hydrochloric acid.*
Weigh 12.5 g hydroxylamine into a 250 ml volumetric flask. Make up volume with 2 M hydrochloric acid. This amount of solution is sufficient for two complete runs.

*R 2: Buffer at pH 5.0*
Weigh 272.2 g sodium acetate into a 1 litre volumetric flask. Make up volume with water. Add 2 M acetic acid to this solution until pH 5.0 is reached. Add 1 ml of wetting agent (Brij 35) per litre of the buffer. Discard the solution at first sign of turbidity.

**Technicon Instruments Inc.*

